# A mixed methods analysis of quality of life among late-life patients diagnosed with chronic illnesses

**DOI:** 10.1186/s12955-017-0797-3

**Published:** 2017-11-16

**Authors:** Monica S. Frazer, Patrick Mobley

**Affiliations:** 1 Senior Researcher, Optum Division of Health Economics and Outcomes Research, 11000 Optum Circle, Eden Prairie, MN 55344 USA; 2 Data Scientist, Sentience Data and Analytics, Honeywell, Inc., 12001 Hwy 55, Plymouth, MN 55441 USA

## Abstract

**Background:**

Quality of life (QOL) is an important consideration for people living with advancing chronic conditions. Palliative care providers speak about how, despite physical decline in late life, many patients report growth and meaning in other domains. This mixed methods study uses QOL survey responses to explore domain trajectories and interview data to explore how patients with advancing chronic conditions experience distinct QOL domains.

**Methods:**

The study sample includes 156 now-deceased participants who completed the FACIT-Pal quarterly, and 40 (10 now-deceased) participants who discussed QOL in an interview. Mean subscale scores were plotted over participants’ last 18 months to reveal QOL trajectories. Interview data were analyzed to reveal how participants’ experience, actions and cognitive processes influenced QOL scores.

**Results:**

Physical and functional subscale ratings show gradual decline. Emotional QOL maintains with a small dip 2–3 months before death, and social QOL ratings improve in participants’ final 3 months. Participants create and strengthen relationships that help them better manage health and receive instrumental and emotional support; seek activities in which they can find joy, meaning, and purpose; and support cognitions through which patients accept and communicate about illness, and emphasize positives.

**Conclusion:**

QOL domains exist in different trajectories. Despite physical and functional decline, participant ratings of emotional QOL maintain and ratings of social QOL improve at end of life. Understanding the processes through which participants countered declining QOL may help providers identify how to best support and promote improved QOL for patients during their final months.

## Background

Advances in medical treatment have resulted in a growing demographic of individuals who are living longer with chronic illnesses. Two-thirds of adults over 65 have at least two chronic conditions and nearly one-quarter of Medicare patients have 5 or more [1, 2]. Comorbid chronic conditions are associated with increased care needs, functional limitations, and more negative health outcomes [3]. Quality of life (QOL) is recognized as increasingly important for patients with chronic conditions as physical abilities diminish [4–8]. The association between QOL and comorbid chronic disease is complex and depends upon disease type, disease combinations, and non-physical factors. Using primarily cross-sectional methods, researchers investigating specific diagnoses and disease constellations have found generally negative effects on QOL, although specific findings and operationalization of QOL vary [4, 9–12]. Consideration of disease burden has revealed a more direct negative effect upon QOL [9, 13–15]. Psychological and social resources have been found to help individuals preserve QOL [7, 15]. However, most QOL research has not included consideration of disease stage [10].

As populations live longer and comorbidity becomes more prevalent, there is a need for more research to illuminate how individuals with advancing chronic illnesses live their final months [15, 16]. Over time, the effects of illness accumulate, options change, and ultimately social and emotional transitions associated with impending death must occur. Longitudinal research that allows for identifying trajectories offers potential for better understanding the link between disease stage and QOL. Trajectories that show how functional decline differs based on disease and cause of death have proven useful in understanding and anticipating patient needs [17, 18]. Trajectories revealing how patients’ social, psychological and spiritual needs mirror and diverge from physical needs, depending upon diagnosis and seminal illness events during patients’ final 12 months, can help providers anticipate needs and assist in planning [19]. Understanding how distinct QOL domains change over time could also assist providers in predicting the likely needs of patients so support is sensitive and more effective [20].

To date, the studies presenting end-of-life trajectories have used qualitative data. The present study adds to research about QOL trajectories by using qualitative and longitudinal quantitative data to explore whether QOL domains exist in different trajectories and why. The research questions are: Among late-life patients with chronic illness, how might ratings of QOL domains differ over time? What influences how participants assess QOL? This research ultimately aims to inform care providers who want to support better life quality for patients in late life.

## Methods

This mixed methods research includes quantitative and qualitative data within a single analytical plan to build upon the strengths in each method and generate results that more fully and deeply answer research questions [21, 22]. A parallel convergent design allows examination of both data types in concurrent fashion [23]. The quantitative component allows visual mapping of aggregated scores for participants’ reported QOL, while the qualitative component allows understanding of the processes and experience that influence QOL ratings [24].

### Participants

This sub-study includes 156 now-deceased participants who completed a quarterly QOL survey for at least 18 months before death and 40 patients (10 now-deceased) who discussed QOL in interviews (Table 1). This study is part of a larger project called LifeCourse that tests a whole-person psychosocial approach for patients during their last two to three years. Study participants have a primary diagnosis of advanced heart failure, stage 3 or 4 cancer, or advanced dementia. Lay healthcare workers called care guides meet with participants and their family caregivers at home for the duration of their care. Care guides work within interdisciplinary teams [25] to support person-centered, palliative practices earlier in the disease process [26, 27]. Care guides use a visit protocol that includes questions about 11 domains of personhood and are trained to conduct advance care planning [28–30]. Care guides help patients articulate goals and provide referrals or link patients with needed services. Whole person information is documented in the patients’ electronic health record to promote a broad awareness of the patient’s circumstances and goals. The study setting is a large Midwestern metropolitan healthcare system. Approval to conduct this study was granted by Quorum institutional review board.

**Table 1:** Participant Characteristics

	Survey Sample *N* = 156	Interview Sample *N* = 40
Age (mean years ± sd)	74.9 ± 13.1	75.6 ± 11.2
Comorbidities (mean # diagnoses ± sd)	5.4 ± 1.6	5.4 ± 1.7
Female	43.0%	45.0%
Caucasian	97.4%	97.5%
Marital Status		
Single, never married	10.3%	7.5%
Married, or civil union	48.7%	57.5%
Unmarried partnership	1.3%	2.5%
Divorced/separated	14.7%	10.0%
Widowed	25.0%	22.5%
Highest Level of Education		
High School or less	38.5%	20.0%
Some college to bachelor’s degree	43.6%	45.0%
Graduate/professional school	16.0%	32.5%
Unknown	1.9%	2.5%
Baseline Living Location		
Home	83.3%	90.0%
Assisted living	11.5%	7.5%
Nursing home	5.1%	2.5%
Primary Diagnosis		
Heart failure	71.8%	72.5%
Cancer	28.2%	10.0%
Dementia	0.0%	17.5%

*Sd* standard deviation

### Data collection

The Functional Assessment of Chronic Illness Therapy-Palliative Care scale (FACIT-Pal) is a 46-item multidimensional measure of general health-related quality of life that targets the management of chronic illness. The FACIT-Pal contains 4 subscales that measure physical, social, emotional, and functional QOL with an added 19-item palliative subscale that explores palliative aspects of care such as relationships, weight loss and dry mouth, decision making capability, and feelings of peace and hopefulness [31, 32]. The palliative subscale is often combined with other scales for a total score. This study uses data from the 4 core QOL subscales only because questions on the palliative subscale crossed domains and were not mutually exclusive from the other 4 subscales. The FACIT-Pal is widely used by health care systems in the US and has been shown to have high internal consistency, reliability, and validity [31]. Study participants were asked to complete the survey quarterly for the duration of their participation. Data used in the present study was collected between October 29, 2012 and September 6, 2016.

Sixty-five participants were identified for interview recruitment. Potential interviewees had completed surveys during the study window, reported a range in healthcare experience, and represented all 3 primary diagnosis categories. The first author called all potential interviewees and 42 accepted the invitation to be interviewed. At the interview, participants were screened to ensure they possessed the cognitive capacity to provide informed consent. The consent form was reviewed and participants had to correctly answer 4 questions about what was explained to pass screening. Two did not pass the screening, leaving 40 interview participants (62% of 65 invited). Interviews with participants were conducted either individually (*n* = 24) or with caregivers present (*n* = 16), based on participant preference. QOL questions included asking participants to describe what a good day is like and what a bad day is like, and probes were used to explore specific QOL domains. Interviews lasted between 90 and 120 min and were conducted by the first author between August 28, 2014 and February 23, 2016. Interviews were audio taped and transcribed. Quotes pertaining to QOL were used as data for this study.

### Analysis

The aim of the analysis was to see how QOL subscales plot during the last 18 months of life and to understand how participants assess their QOL. Survey results were oriented on when participants died, moving back in time. This analysis examines the 4 core FACIT subscales (see Table 2). Mean subscale scores were calculated and plotted over time to reveal trajectories for each QOL subscale. Responses were ascribed a value between 0 (not at all) and 4 (very much). Values were summed and divided by the number of questions on the subscale to determine the score for each patient. The mean scores for each subscale are presented. The emotional subscale contains one less question than the other subscales, so emotional QOL scores were proportionally weighted before mean scores were calculated. The resulting subscales all had a possible score of 0 to 28 making comparisons more intuitive and standardized.

**Table 2:** FACIT Questions by Subscale

Physical	I have a lack of energy
	I have nausea.
	Because of my physical condition, I have trouble meeting the needs of my family.
	I have pain.
	I am bothered by side effects of treatment.
	I feel ill.
	I’m forced to spend time in bed.
Social	I feel close to my friends.
	I get emotional support from my family.
	I get support from my friends.
	My family has accepted my illness.
	I am satisfied with family communication about my illness.
	I feel close to my partner (or the person who is my main support).
	I am satisfied with my sex life (option to not answer).
Emotional	I feel sad.
	I am satisfied with how I am coping with my illness.
	I am losing hope in the fight against my illness.
	I feel nervous.
	I worry about dying.
	I worry that my condition will get worse.
Functional	I am able to work (include work at home).
	My work (include work at home) is fulfilling.
	I am able to enjoy life.
	I have accepted my illness.
	I am sleeping well.
	I am enjoying the things I usually do for fun.
	I am content with the quality of my life right now.

Items responses exist on a Likert scale: not at all (0), a little bit (1), somewhat (2), quite a bit (3), very much (4) with options for don’t know (dk) and not applicable (na)

Qualitative data pertaining to QOL were initially identified by two researchers. The QOL quotes were deductively coded to the 4 FACIT-Pal domains by a single researcher (MF). This coding was reviewed and confirmed by a team of 4 qualitative researchers. Quotes assigned to each subscale were coded using a phenomenological approach aimed at discovering participants’ cognitive processing and reported actions that affect life quality [33]. To mix methods, quotes within each domain were compared to FACIT-Pal questions to reflect survey content and identify additional experiences. Interview data prompted new ways to look at survey results, which generated additional questions that could be explored within the interview data. Findings within each domain were ultimately grouped into themes. This back and forth between data sources is the hallmark of the parallel convergent design.

Both authors were employed by the health care organization that hosted the larger study, working as a senior scientist (MF) and data analyst (PM) within the organization’s research division. Neither had regular participant contact but both had personal experience with loved ones who died from comorbid chronic illness. The interviewer attempted to use her personal experience to connect with study participants and explore responses to a level of depth that allowed more nuanced understanding of QOL experience.

## Results

**Fig. 1:**
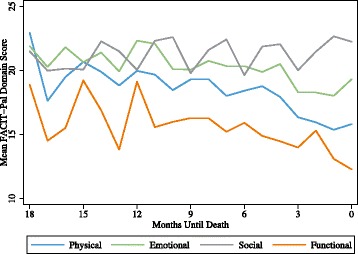
Mean Scores for QOL Domains during Participants’ Last 18 Months of Life (*n* = 156)

**Fig. 2:**
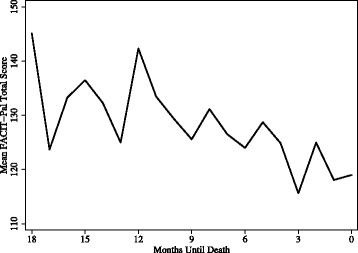
FACIT-Pal Total Mean Scores during Participants’ Last 18 Months of Life

**Fig. 3:**
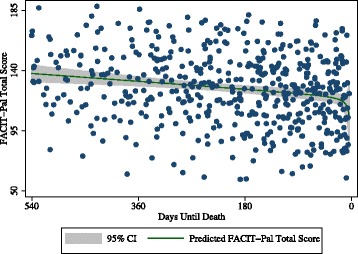
Scatterplot of FACIT-Pal Scores in Participants’ Last 30 Months

The plotting of participant subscale scores in distinct QOL domain trajectories reveals that QOL domains move independently of each other, following unique trajectories over participants’ last 18 months (Fig. 1). Oscillations such as those visible in the social subscale are largely due to cohort effects of receiving surveys every three months. Subscale trajectories reveal distinctions in experience that are not apparent when plotted as the full scale (Fig. 2). A scatterplot of total scores reveals that individual scores vary considerably (Fig. 3), but trend downward overall despite month to month turbulence. Interviews with participants revealed actions and cognitive processes that had an impact upon QOL ratings in each subscale.

### Physical QOL

In the last 18 months of life, participants, on average, rated their physical QOL higher than their functional QOL, and lower than social and emotional QOL. Participant comments reveal significant changes in how participants live day to day because of their health condition, and give insight into how they maintain health and preserve important activities.

Items on the FACIT-Pal physical subscale ask primarily about physical symptoms. Participants endorsed symptoms such as pain, lack of energy, and nausea, and talked about symptoms not covered in the survey such as vertigo, wobbliness, numbness, and confusion. Participants also discussed how the sum of physical effects from their condition and treatment change their daily existence (“*Some of the side effects from the drugs, that’s definitely altered my life. …the effects from the surgery mean that I really can’t do some of the things that I used to do physically*”). Physical effects reduced participants’ ability to conduct simple tasks (“*Every stitch of clothing except my bra was on backwards this morning. I had to re-dress myself*”) and limited activities such as travel (“*It’s hard to vacation and have a good time if you’re in pain. If you’re not feeling well, it’s tough*”), exercise (“*I was going to water aerobics, but now I can’t with my feet*”) and hobbies (“*Fishing-I quit two years ago. I sold my boat. It was getting too hard to get it in the water and back out*”).

Participant comments reveal the complexity of their health conditions due to comorbidity (“*I also have, as I say, three or four chronic diseases. I can live the any one of them. If two or more of them rear their ugly heads at once, I’d be in trouble*”) and how participants must simultaneously manage multiple diseases (“*I deal with hepatitis C...it’s a silent killer...I deal with emphysema in my right lung, which doesn’t affect me right now. My legs, I have six veins plugged up...if I don’t take care of my legs, they’ll take me out. The emphysema will eventually take me out.*”). A spouse explains how her husband’s complex condition requires constant vigilance,

“*Just like that [snaps fingers], you know, it can get worse really quick. I don’t think a lot of people realize, when you have people that have been really sick, you know, you’ve got to really watch, because the same things that for a healthy person wouldn’t affect them, whereas somebody with [his] health, you’ve got to watch really close, because it can turn quickly*.”

One item on the physical QOL scale is a question about respondents’ ability to meet family needs, which can pertain to a wide variety of activities. Coping with illness meant accepting physical changes and coming to terms with a new way of being (“*Between the heart failure and the abdominal stuff, totally reshaping my world. I don’t know. It’s drastically changed what I do and how I do it, my energy level and so much of it is out of my control, I just have to accept it*”) that opened the door to preserving abilities that allowed participants to conduct targeted tasks (“*We gave up our car around a year ago because both of us could drive, but neither one of us could walk well enough to go very far after we got where we were going*”). All participants relied upon family and friends or purchased services to complete routine tasks. Over half reported they had someone come into their homes to clean (“*The cleaning and the wash, we have a girl come in twice a week*”) and a third use Metro Mobility or rely upon others for rides after giving up their car. Many also had medical services in the home (“*When I needed my foot dressed...we arranged for one of the healthcare assistants to come in every day and change the bandage for me*”) that preserved time and energy for preferred activities. Some participants got help with basic tasks like dressing and hygiene (“*Back to my bra again...she’ll help me and then she’ll help me get my shirt on too*”).

Behind the experience of physical symptoms are actions people take to manage their care and alleviate negative effects. Sixty percent of interview participants lived with a spouse or partner. They supported each other in reciprocal partnerships (“*We’re a fine oiled machine as far as helping each other*”) in which health needs were accommodated without question or complaint (“*She’s always been there to help drive me, especially push me...in a wheelchair, because me going to some places is very hard breathing-wise, because I have to stop every 10, 5 steps*”). Managing illness and completing tasks was done within an unconditional family enterprise built upon partners’ love and commitment to each other.

Participants also created supportive partnerships that helped them better understand their health conditions and options. Seven participants talked about having a friend or relative with insider knowledge about healthcare who provided advocacy, education, and coached participants on how to work with providers. One couple described how their daughter-in-law, who is a nurse, helped,

Husband: “*She knows there’s a lot that I don’t understand...like, especially if it’s hard or technical...she’s keeping track to that pretty good for me*.”

Wife: “*So she’d just as soon go and listen…she’ll ask the doctor if [husband] didn’t, “can you tell him more about this?” Or when we get home, she’ll tell us about it. I get my book out—they always give me a book on that heart. She’ll show me in there what he’s talking about, this valve and this thing, here’s your trouble*.”

Six participants spoke about a profound trust they placed in doctors who they perceived extended a level of extra effort, as exemplified in the following,

“*I give a lot of credit to [my doctor]. He’s the one that tells me where to go to, and who to see, and everything else. [He] is like my big brother, or maybe an uncle. I mean, he’s so caring. I mean, he calls me on his way home...I can talk to him about anything…, I feel so complete…He’ll tell me even things that I don’t know. That’s how much he cares, and so I guess I’m one of his favorite patients, so my brother says, because he come down to see me in the hospital*.”

Five participants worked in healthcare organizations themselves. Their professional affiliation not only helped participants better understand their illness and treatment, it could garner a different level of care from providers,

“*I kind of had a different thing going at the hospital because I wasn’t – yes, I was a participant, but I wasn’t* *just* *a participant, I was like one of them...These nurses would come in, and they would talk to me, and they were like, ‘Don’t let these cardiologists push you around...make them explain why they want to do this, or what they’re doing…Don’t let them just run over you!’ One of them even came in one evening with a pad of paper and a pen so we could brainstorm questions and then write them down so I wouldn’t forget them*.”

Participants talked about how they face complex physical challenges that had drastic impacts upon their daily lives. Despite those challenges, participants accepted their health conditions, accomplished what they could, and relied upon others to help them understand illnesses, navigate care, and complete daily tasks. Ratings of physical QOL reflect the positive strategies and assistance upon which participants could rely in the face of physical challenges.

### Social QOL

Ratings of social QOL are unique in that they defy the downward trend seen in other subscales. The social QOL trajectory maintains over time compared to the other subscales, before ratings improve in the final 3 months of life. Survey questions make a distinction between social and emotional support, but participants talk about support more generally. While loneliness was expressed (primarily related to loss of loved ones), participant comments reveal rich social interactions with family, friends, and professionals who show support through frequent interactions. When asked about what makes for a good day, time with others was consistently identified as an essential element (“*A good day for us, too, is we’re meeting with friends. We’re having a good time. I’m making [my husband] dance. That’s a great day for me. I might talk to my kids during the day, see my girlfriend. Very fulfilled*.”) Although questions about sexual intimacy were not included in the interview protocol, 3 husbands shared feelings of sadness and loss related to decreased intimate time with their partner.

As family and friends come to understand and accept the severity of their loved one’s health condition, they made time to be with and do things for their loved one (*My “family has been aware of that situation and so they’ve gone a little bit further in trying to make sure that, you know, that they can help out whenever they can*”). Time spent related to illness could offer an opportunity to enhance relationships (“*When you’re in pain and you’re uncomfortable, you get to know somebody pretty darned well. When you’re trying to help them or whatever. Yeah, I think it’s deepened our relationship*”). Participants who did not have close family relationships created their own family of friends. Interactions with neighbors and service providers were noted as important social interactions.

While some participants freely shared information about their illness with adult children, others were careful not to burden their children with knowledge about their health condition (“*Any worries I have, I really don’t share those with my kids so much. I don’t really need to place that burden on them*” and “*My kids have no idea how close I am to death*”) or ask too much of their loved one’s time (“*I try not to burden them, try not to say I have to go this many places and do this many things immediately*”). As illness increasingly dictates choices and actions, one of the places where control can still be exercised is in what information is shared and with whom. Participant comments did not reflect consideration of their loved one’s experiences when they were not informed about diagnoses or prognosis.

Participants expressed that living through illness with a partner brought a new dimension to their relationship. Both married and unmarried partners described an intense enterprise in which they helped and supported each other. Social QOL is unique in that it is the only subscale trajectory in which mean ratings improve over time. Contact with and support from others occurs within the serious illness experience, promoted by knowledge and acceptance of illness, supported positive feelings about social QOL.

### Emotional QOL

Emotional subscale ratings reveal that participants enjoy relatively high emotional QOL in spite of physical and functional limitations. Emotional QOL is rated much the same as social QOL until about 10 months prior to death. Ratings remain even between 10 and 4 months, and then decline in the final 3 months. Participants expressed that illness takes a toll (“*Pain is a bad thing. It makes you feel awful. It’s emotionally and physically draining*”). Expressed emotions included sadness (“*When you start having all these issues, you start to see where people, where the depression and suicide factor comes in. It’s like, I’m so tired of the fight and so tired of the pain…I just want to relax. I just don’t want to hurt any more. I just don’t want to put up with the stress anymore*”) and worry (“*I start to worry if I have a cough. I start to worry that, oh my gosh, [my cancer is] growing. Anything that is unusual, you go to the darkest place*”). Participants also expressed feelings of frustration (“*You’re reliant on other people. You want to be able to do stuff for yourself without having to ask somebody for help all the time*”) and anger (“*I just truly hate sitting and watching her doing things I should be able to do...that upsets me*”). Loneliness was also commonly experienced, (“*I am so alone. I can’t believe it that I don’t have anybody, not even my brother, to count on anymore*”) reflecting the loss of loved ones, difficulty getting out, not feeling up to visits, and the isolating effects of depression. Participants did not discuss feeling hopeless. In fact, most expressed acceptance of illness and a pragmatic approach to daily living.

Despite feeling difficult emotions, participants worked to maintain a positive outlook (“*If you have the attitude that you’re going to live as long as you possibly can and so, do most of the things that you want to do, you should do it. You just have to have a good attitude*”). Participants talked about purposeful thoughts (“*I’ve had to learn to step back for a minute and wait a minute, and be rational about it*”) and actions they took to distract them from feeling bad (“*My legs start shaking and they ache…if they start messing up, I just go down there and start doing [my woodworking], and forget about them. It just takes my mind off of it*”). Participants compared themselves to others to assess their coping (“*Her dad has problems almost as bad as mine. However, he doesn’t acknowledge them, whereas I know what my problems are and because of that, I better do what the doctors tell me...maybe just his normal attitude is more negative than mine*”).

Most participants made efforts to express gratitude for the supports they received and identify positive aspects of their experience (“*Each of us says, ‘Any day not in the hospital is a wonderful day!’ and it really is…We’re in the fourth quarter of our life, so it could end any time, but as long as we’re comfortable, have our wits about us and I have my sense of humor, I choose to go on*”). One participant living with stage IV cancer stated,

“*I’m truly fortunate because it could be a lot worse for me. I don’t have pain. I don’t have chaos in my life. I don’t have a lot of worries about financial, am I going to be able to pay for my drugs. I’m totally mobile. I can still do everything that I want to do. I grow. I grow every day, and even though I don’t have long-term goals, I am very satisfied with my life and where I am*.”

Struggles shared by participants reveal a range of felt emotions as they faced ongoing pain and loss of ability. Participants worked hard to focus on the positive in effort to boost emotions. The decline in emotional QOL ratings at 4 months before death suggests that this is a point where specific support for emotional QOL could be beneficial. Having others with whom participants can express emotions and not feel alone was recognized as important.

### Functional QOL

The functional QOL trajectory reveals that participants consistently rate their functional QOL lower than other subscales in their final 18 months, and that the decline in ratings is similar to that of physical QOL. Questions on the functional subscale explore work, enjoyment and contentment, acceptance of illness, and sleep quality, and low scores reflect the ways in which illness eats away at how participants experience daily life.

Work was discussed in a broad sense by interview participants. Nine participants were employed at the time of their interview, despite their health condition. Illness effects challenged participants’ ability to get to work and do their job (“*I could barely walk up the stairs. I was that bad*” or “*He would get out of bed and he would crawl into the bathroom, hold himself, standing up crying from the bathroom vanity, crying, to get ready for work*”). Participants kept employment because it brought purpose and distraction from struggles, they had a strong work ethic, and because wages and insurance were necessary to pay for medical care. Participants who were not employed ‘worked’ on hobbies in which they created things like furniture, art, handmade gifts, wrote books, or researched genealogy. Ten participants sought out volunteer activities that made them feel useful (“*I was a high school principal and I helped him prepare for his tests, his state tests...I felt that I really could make a substantial, fill a substantial gap for him*”) and enabled meaningful relationships (“*I am tutoring a Russian speaking woman on English...It started out as a student-teacher relationship. It’s now become, it’s grown into much more of a family relationship. We feel she’s like a daughter we never had*”). These activities brought satisfaction and joy, especially when activities provided mutual benefit.

Participant comments reveal acceptance of illness and current abilities (“*I’m walking the last leg, and that’s fine. I have no fear of death whatsoever, so I’m not pondering or having that kind of hanging over my head*” and “*It would be great if I was in better health, but this is the reality and so, yeah, you make the best of where you’re at*”). Comments reveal that participants were not happy about their health condition, but they knew they wouldn’t change it or be cured. Getting quality sleep was a challenge faced by all.

Participants were frank about their limitations and accepting their fate. Participants defied significant physical challenges so they could work and engage in enjoyable, meaningful activities. Although participants accepted limitations and sleep difficulties, the constant need to adjust and overcome challenges meant that functional QOL was rated more poorly than other domains.

## Discussion

Results from this study reveal how mean QOL subscale ratings vary over time for participants in their last 18 months, and that despite diminishing physical health and function, participants’ emotional QOL ratings stay relatively the same and social QOL ratings are higher as death nears. In the face of complex and burdensome diagnoses, participants used cognitive processes like accepting their fate, comparing their situation to others, focusing on the positive aspects of their lives, stemming negative thoughts, and expressing gratitude to keep emotional and social QOL ratings relatively high. Actions included seeking out meaningful and enjoyable activities, finding a knowledgeable health advocate, asking for or hiring help to complete selected tasks, and making changes to accommodate illness.

There are two contextual elements that deserve consideration. Study participants were part of an intervention in which lay healthcare workers regularly visited to promote palliative practices earlier in disease than typically occurs. It is possible that monthly visits from a care guide could have a positive impact upon emotional QOL as regular contact reduced feelings of isolation. Secondly, findings from the present study reveal different outcomes than Murray and colleagues (2007) found when plotting trajectories for physical, social, psychological and spiritual needs. While both studies find downward trends for the physical domain, the present study finds different and more divergent trajectories, especially for the emotional/psychological and social domains. Response shift, the process through which individual values and standards change in response to health events, has been identified as an important influence on how people rate QOL [34, 35]. Over time, judgments become more lenient and measurement standards change [36]. Response shift effects could possibly account for differences between findings because response shift effects have been identified as a concern related to survey responses and Murray’s findings are based on qualitative interviews. The thoughts shared by participants in the current study illustrate how they adjust expectations and choose enriching activities that support a more positive QOL assessment. Participant comments also reveal factors (beyond FACIT-Pal questions) that affect QOL ratings in addition to the intra-personal mechanisms of response shift.

This study presents an early exploration of trajectories to better understand late life experience for individuals living with chronic illnesses. More research is needed, especially studies that address limitations. Study participants were largely white and well educated, so inclusion of a more diverse participant pool is needed. One-quarter of interview participants died during this study, so most interviews occurred prior to participants’ final 18 months. While challenging, more interviews that occur closer to the participants’ death are needed. This research includes 4 QOL domains, and most notably does not include a measure of spirituality which has shown to have protective influence at end of life [37], and complex associations with QOL [38–40].

## Conclusion

Study findings can inform professionals who work with late life patients to encourage adaptations [41] and promote self-efficacy [42] that have been shown to positively affect QOL. Physical QOL was improved when participants found an advocate who helped them fully understand their health condition and navigate healthcare systems, and by enlisting or purchasing help with physical tasks. Help from others allowed participants to remain at home and save energy for other activities. Functional QOL was improved when participants accepted current limitations, engaged in employment as able, and pursued hobbies and volunteer activities. Helping participants see positive aspects in their experience and coaching participants in techniques to combat negative emotions can stem emotional QOL diminishment. Care providers can support improvements in social QOL by encouraging discussion about serious illness with loved ones, reliance upon others as participants feel comfortable, and encouraging social time with others. Participants talked about the ways in which they successfully shaped their thinking and took action to improve their daily lives. Professionals can promote these strategies in work with others in effort to keep QOL as positive as possible.

## Abbreviations

FACIT-Pal: Functional Assessment of Chronic Illness Therapy-Palliative Care scale
QOL: Quality of life
